# *LncRNA KCNQ1OT1* regulates *microRNA-9*-LMX1A expression and inhibits gastric cancer cell progression

**DOI:** 10.18632/aging.102651

**Published:** 2020-01-08

**Authors:** Li Feng, Huanqin Li, Fan Li, Songhua Bei, Xiaohong Zhang

**Affiliations:** 1 Endoscopy Center, Minhang Hospital, Fudan University, Shanghai, China

**Keywords:** gastric cancer, *lncRNA*, *KCNQ1OT1*, *microRNA-9*, LMX1A

## Abstract

LMX1A (LIM homeobox transcription factor 1α) is a tumor suppressor protein. Our previous study has shown that *microRNA-9* (“*miR-9*”), being upregulated in human gastric cancer (GC), targets LMX1A to promote GC cell progression. Through searching *long non-coding RNA* (*LncRNA*) database, we identified that *LncRNA KCNQ1OT1* is the *competing endogenous RNA* (ceRNA) of *miR-9. KCNQ1OT1* putatively targets *miR-9*. Its level is downregulated in human GC tissues. In AGS cells and primary human GC cells, forced overexpression of *KCNQ1OT1*, by a lentiviral construct, induced *miR-9* downregulation and LMX1A upregulation. Furthermore, *KCNQ1OT1* overexpression inhibited GC cell survival, proliferation, migration and invasion, but inducing apoptosis activation. Contrarily, *KCNQ1OT1* silencing, by targeted siRNAs, induced *miR-9* accumulation and LMX1A downregulation. Consequently, GC cell proliferation, migration and invasion were enhanced. Importantly, *KCNQ1OT1* overexpression or silencing was ineffective in LMX1A knockout AGC cells. Taken together, *KCNQ1OT1* inhibits GC cell progression via regulating *miR-9* and LMX1A expression.

## INTRODUCTION

Gastric cancer (GC) is a common cancer, causing significant human mortalities each year [1]. In the past decades, due to the developments of sanitation, control of *Helicobacter pylori* infection and GC screenings, its incidence is declined [2, 3]. However, the prognosis for the metastatic, recurrent, and other advanced GCs is not optimistic [1]. Identification of novel therapeutic targets and biomarkers for GC is extremely important [4, 5]. It is the research focus of our group [6, 7].

LMX1A (LIM homeobox transcription factor 1α) is the primary member of LIM-homeodomain conserved family transcription factors [8]. LMX1A regulates a number of physiological and cancerous behaviors [8]. LMX1A is downregulated in GC [7] and many other cancers [9, 10]. Our group has shown that LMX1A exerted tumor-suppressive functions in GC cells [6, 7, 11]. We found that LMX1A inhibited c-Myc expression to exert tumor suppressive function in GC cells [6]. Also, LMX1A inhibited GC cell metastasis by inhibiting beta-catenin-dependent genes [11].

microRNAs (miRNAs) are small, noncoding RNAs, regulating target gene expression by suppressing mRNAs translation and/or inducing their degradation. Our previous study has identified a LMX1A-targeting miRNA: *microRNA-9* (*miR-9*). We show that *miR-9* selectively targets and negatively regulates LMX1A to promote GC cell progression [7]. In human GC tissues, *miR-9* upregulation is correlated with LMX1A downregulation [7]. The mechanism of *miR-9* upregulation in human GC is still elusive.

*Long non-coding RNAs* (*LncRNAs*) are a large family of conserved *non-coding RNAs* (*ncRNAs*) with over 200-nucleotide long [12–14]. *LncRNA* can decrease target miRNA expression by acting as their sponges [15]. *LncRNAs* regulate key cellular activities, including genomic imprinting, cell proliferation and survival, cell cycle control and differentiation as well as apoptosis and transformation [12–14]. Accumulative studies have confirmed that dysregulation of *LncRNA* plays a pivotal role in the progression of GC [15, 16] and other human cancers [17, 18]. One aim of this study is to identify possible miR-9-targeting *LncRNA*, regulating LMX1A expression and GC cell functions.

*LncRNA* *KCNQ1OT1*, or potassium voltage-gated channel subfamily Q member 1 (Kcnq1) overlapping transcript 1, is an imprinted antisense *LncRNA* [19, 20]. It is an overlapping transcript of Kcnq1, locating at Kcnq1 loci on chromosome 11p15.5, being exclusively transcribed from the paternal chromosome [19, 20]. Recent studies have shown that *KCNQ1OT1* dysregulation participates in carcinogenesis and progression of human cancers [21, 22]. Its expression and potential functions in GC are largely unknown. In the present study, we will show that *LncRNA* *KCNQ1OT1* inhibits GC cell progression possibly via regulating *miR-9* and LMX1A expression.

## RESULTS

### *LncRNA KCNQ1OT1* is downregulated in human GC tissues

It is possible that *miR-9* upregulation in GC (see our previous study [7]) could be due to downregulation of certain *LncRNAs*. As they could function as the ceRNAs of *miR-9*. To explore this possibility, the human *LncRNA* database, LncBase (Predicted v.2), was consulted to identify possible *miR-9*-targeting *LncRNAs*. These potential *LncRNAs* were further verified by other *LncRNAs*/*miR* databases, including StarBase and miRbase. The bioinformatic analyses identified that one *LncRNA*, *KCNQ1OT1*, putatively targeted *miR-9*. Its expression in GC tissues was tested next. Total RNA was extracted from fresh GC tissues and the adjacent normal epithelial tissues from twelve (12) primary GC patients [7]. *KCNQ1OT1* was determined by qPCR assay, using the previously-described primers [23].

**Figure 1:**
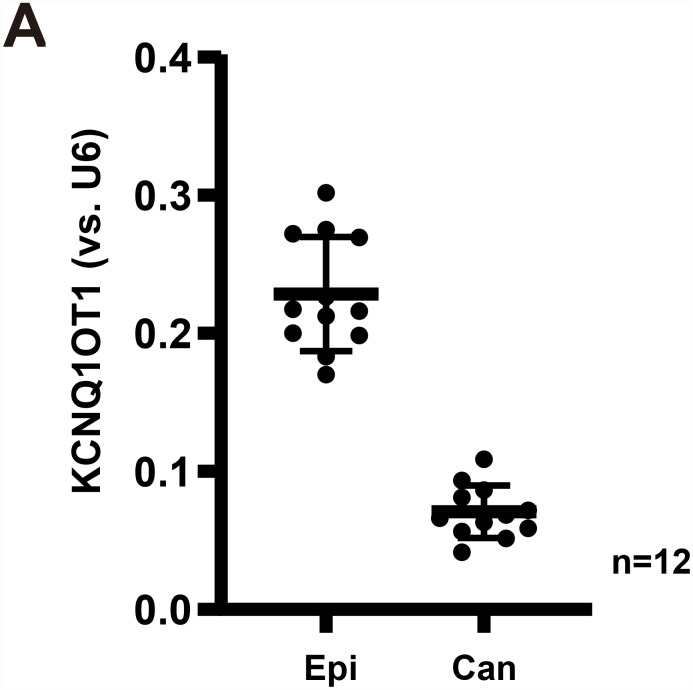
***LncRNAKCNQ1OT1* is downregulated in human GC tissues.** Expression of *LncRNA* *KCNQ1OT1* (**A**) in twelve (12) different human gastric cancer (GC) tissues (“Can”) and matched surrounding normal epithelial tissues (“Epi”) was shown, results were normalized to *U6 RNA*. All values were expressed as the mean ± standard deviation (Same for all Figures). *P <0.05 *vs.* “Epi” tissues.

The results show that *KCNQ1OT1* levels are significantly downregulated in cancer tissues (“Can”) (Figure 1A), as compared to its levels in the surrounding epithelial (“Epi”) tissues (Figure 1A). Therefore, in GC cancer tissues *KCNQ1OT1* downregulation correlated with *miR-9* upregulation (see the previous results from same set of tissue samples [7]).

### Forced overexpression of LncRNA *KCNQ1OT1* induces *miR-9* depletion and LMX1A upregulation in AGS cells

**Figure 2:**
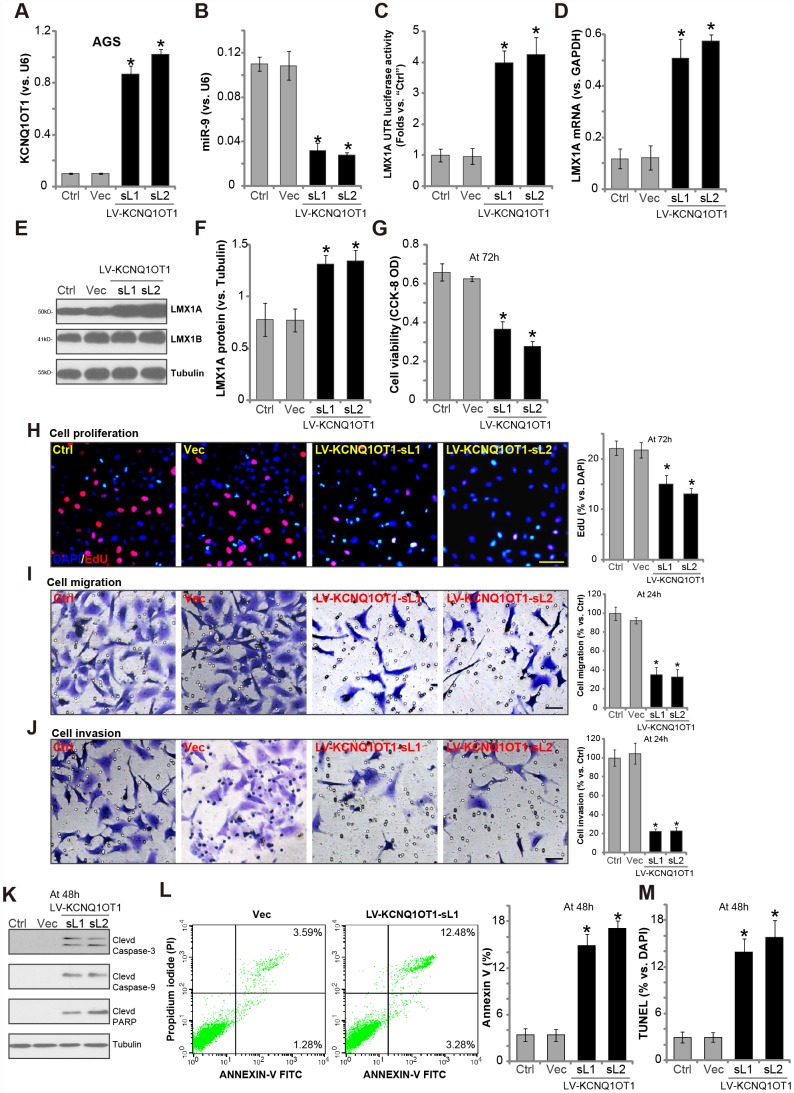
**Forced overexpression of *LncRNAKCNQ1OT1* induces *miR-9* depletion, LMX1A upregulation and AGS cell inhibition.** AGS cells were infected with *KCNQ1OT1*-expressing lentivirus (“LV-KCNQ1OT1”) or scramble control vector lentivirus (“Vec”) for 24h, following puromycin selection two stable lines (“sL1/sL-2”) with LV-KCNQ1OT1 established, expression of *KCNQ1OT1* (**A**), *miR-9* (**B**) and *LMX1A mRNA* (**D**) were tested by qPCR assays; The *LMX1A* 3’-UTR luciferase activity was shown (**C**); Expression of the listed proteins in total cell lysates were tested by Western blotting assay (**E**, results quantified in **F**); Cells were further cultured for the indicated time periods, cell survival and proliferation were tested by CCK-8 assay (**G**) and EdU staining (**H**), respectively; Cell migration and invasion were tested by “Transwell” (**I**) and “Matrigel Transwell” assay (**J**), respectively; Cell apoptosis was tested by Western blotting (testing apoptosis-associated proteins, **K**), Annexin V FACS (**L**) and TUNEL staining assay (**M**). The exact same number of viable cells of different genetic treatment were plated initially (at 0h) for the functional assays (Same for all following Figures). “Ctrl” stands for the parental control cells (Same for all Figures). For each assay, n=5. *P <0.05 *vs.* “Vec” cells. Experiments in this figure were repeated five times, and similar results were obtained. Bar=100 μm (**H**–**J**).

In order to test the potential effect of *LncRNA* *KCNQ1OT1* on *miR-9-*LMX1A expression, a lentiviral GV248 construct encoding *KCNQ1OT1* promoter (“LV-KCNQ1OT1”) was transfected to AGS cells. Following selection by puromycin, two stable cell lines (namely “sL1/sL2”) were established. Testing *KCNQ1OT1* expression, by qPCR assays, demonstrated that mature *KCNQ1OT1* levels increased over 8 to 10 folds in the stable cells (Figure 2A). Significantly, with *KCNQ1OT1* overexpression *miR-9* levels were dramatically downregulated (over 60–70%, Figure 2B), resulting in increased *LMX1A* 3′-UTR luciferase activity (Figure 2C). Moreover, *LMX1A* mRNA (Figure 2D) and protein (Figure 2E and 2F) levels were significantly increased in AGS cells with LV-KCNQ1OT1. Therefore, *KCNQ1OT1* overexpression downregulated *miR-9*, leading to upregulation of its target LMX1A in AGS cells. The lentiviral control vector (“Vec”) had no significant effect on *KCNQ1OT1*-*miR-9*-LMX1A expression in AGS cells (Figure 2A–2F).

### Forced overexpression of *LncRNA* *KCNQ1OT1* inhibits AGS cell progression *in vitro*

LMX1A regulates a number of physiological and pathological processes [8]. It is considered as a tumor suppressor [8, 9, 11]. LMX1A downregulation is detected in GC and many other cancers [9, 10]. Our previous study has shown that LMX1A overexpression can inhibit AGS cell progression *in vitro*. We therefore analyzed the functional consequences of *KCNQ1OT1* overexpression.

Results showed that AGS cells with LV-KCNQ1OT1 presented with reduced cell viability (vs. control cells, Figure 2G). Furthermore, *KCNQ1OT1* overexpression inhibited EdU incorporation (Figure 2H) in AGS cells, indicating proliferation inhibition. “Transwell” and “Matrigel Transwell” assay results, in Figure 2I and 2J, demonstrated that ectopic overexpression of *KCNQ1OT1* potently inhibited AGS cell migration and invasion *in vitro*. Additionally, apoptosis activation was detected in *KCNQ1OT1*-overexpressed AGS cells, evidenced by cleavages of caspase-3, caspase-9 and poly adenosine diphosphate (ADP) ribose (PARP) (Figure 2K), as well as increased Annexin V staining (Figure 2L) and nuclear TUNEL ratio (Figure 2M). The lentiviral control vector (“Vec”) had no significant effect on the function of AGS cells (Figure 2G–2M).

### Forced overexpression of *LncRNA* *KCNQ1OT1* induces miR-9 reduction and LMX1A upregulation in primary human GC cells

**Figure 3:**
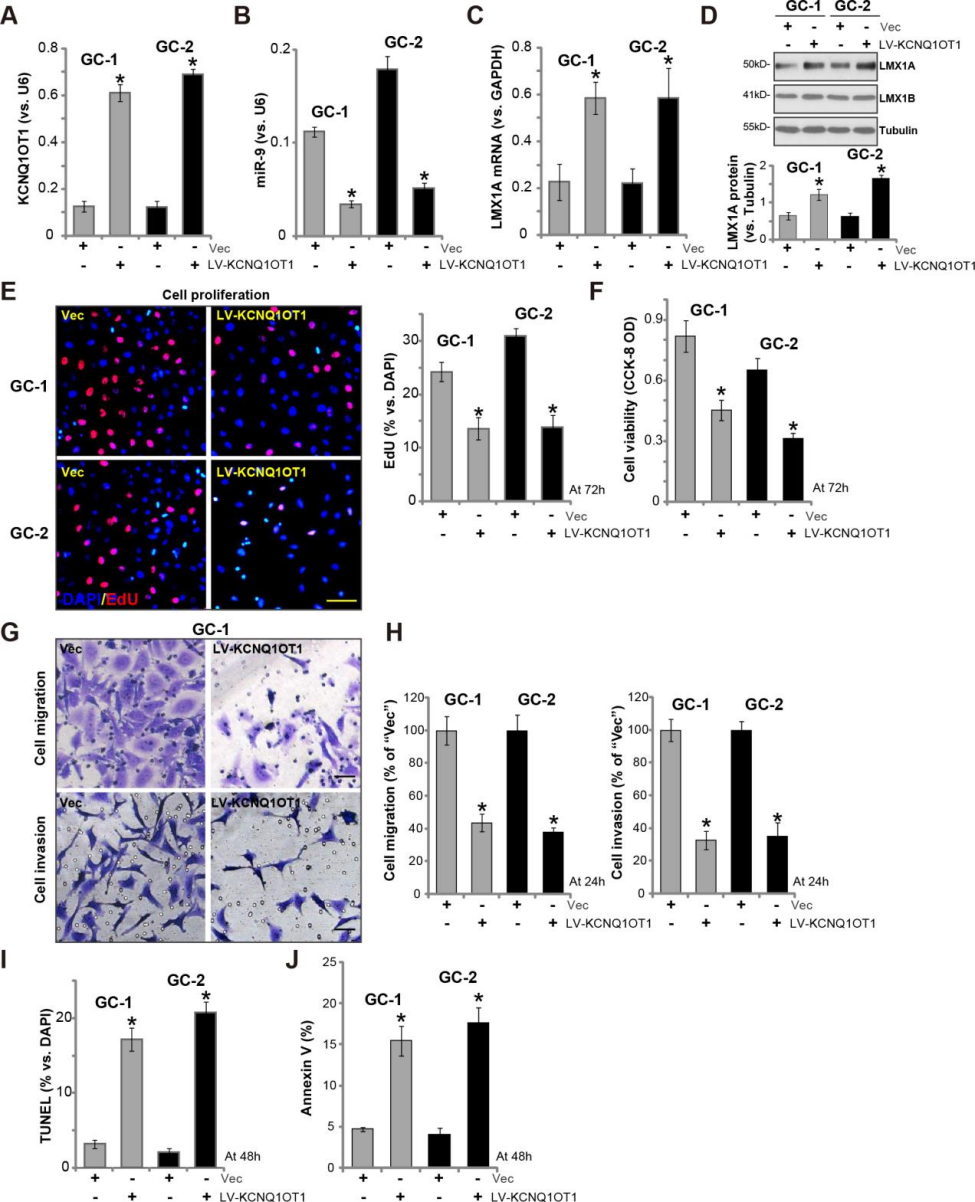
**Forced overexpression of *LncRNAKCNQ1OT1* induces miR-9 reduction and LMX1A upregulation in primary human GC cells.** The primary human GC cells (“GC-1/GC-2”) were infected with *LncRNA* *KCNQ1OT1*-expressing lentivirus (“LV-KCNQ1OT1”) or scramble control vector lentivirus (“Vec”) for 72h, expression of KCNQ1OT1 (**A**), *miR-9* (**B**) and *LMX1A mRNA* (**C**) were tested by qPCR assay; LMX1A and LMX1B protein expression in total cell lysates was tested by Western blotting assay (**D**, LMX1A protein results were quantified); Cells were further cultured for the indicated time periods, cell proliferation and viability were tested by EdU staining assay (**E**) and CCK-8 (**F**), respectively, with cell migration and invasion tested by “Transwell” and “Matrigel Transwell” assay (**G** and **H**); Cell apoptosis was quantified via the TUNEL staining assay (**I**) and Annexin V-FACS assay (**J**). For each assay, n=5. *P <0.05 *vs.* “Vec” cells. Experiments in this figure were repeated three times, and similar results were obtained. Bar=100 μm (**E** and **G**).

Next, we tested whether *LncRNA* *KCNQ1OT1* exerted similar functions in primary human GC cells. As described previously [7], the primary human GC cells, derived from two independent patients (namely “GC-1/GC-2”), were infected with LV-KCNQ1OT1, which resulted in 5-6 folds increase of *KCNQ1OT1* expression (Figure 3A). On the contrary, *miR-9* levels were significantly decreased (Figure 3B), with *LMX1A mRNA* (Figure 3C) and protein (Figure 3D) levels increased in *KCNQ1OT1*-overexpressed GC cells. LMX1B protein expression was unaffected by KCNQ1OT1 overexpression (Figure 3D).

Functional studies show that *KCNQ1OT1* overexpression by LV-KCNQ1OT1 inhibited EdU incorporation (Figure 3E) and cell viability (Figure 3F) in primary human GC cells. Furthermore, LV-KCNQ1OT1-expressing primary GC cells presented with decreased cell migration and invasion (vs. Vector control cells, Figure 3G, 3H). Contrarily, the increased TUNEL staining (Figure 3I, vs control cells) and Annexin V ratio (Figure 3J) were detected in LV-KCNQ1OT1-expressing primary GC cells, indicating apoptosis activation. Therefore, ectopic KCNQ1OT1 overexpression inhibited viability, proliferation, migration and invasion, but inducing apoptosis activation in primary GC cells.

### *LncRNA KCNQ1OT1* siRNA induces *miR-9* upregulation and LMX1A downregulation, promoting GC cell progression *in vitro*

**Figure 4:**
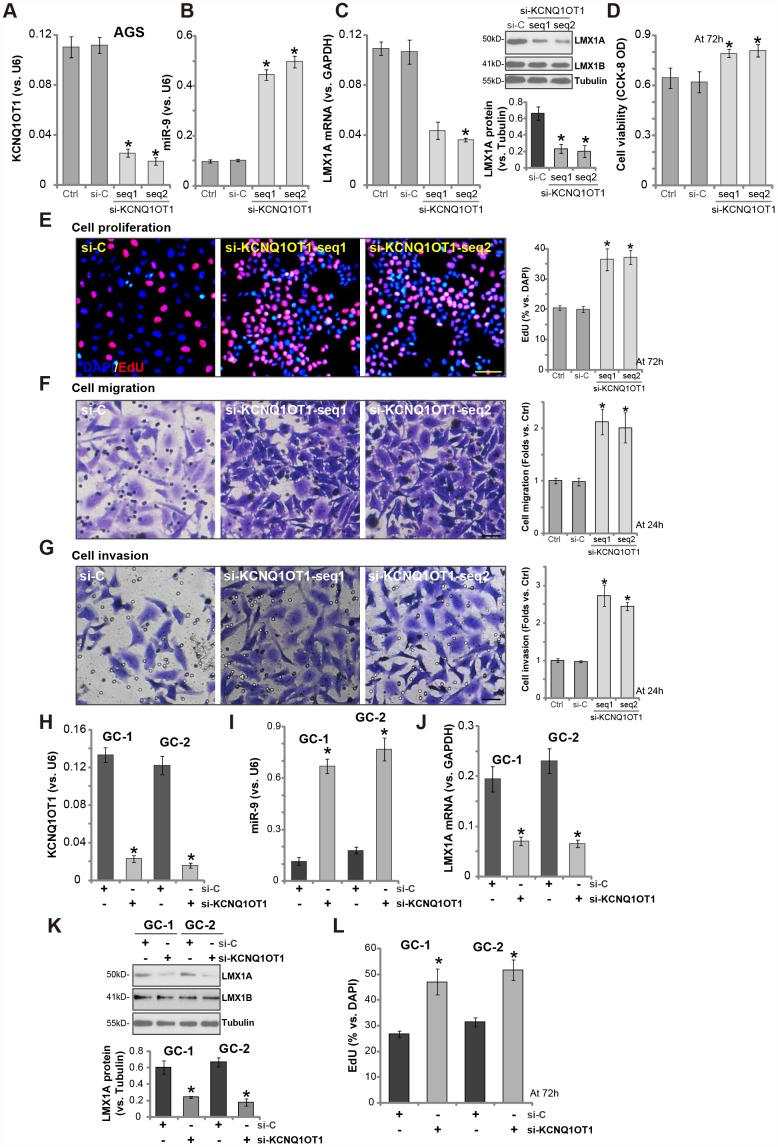
***LncRNA KCNQ1OT1* siRNA induces *miR-9* upregulation and LMX1A downregulation, promoting GC cell progression *in vitro*.** AGS cells (**A**–**G**) or primary human GC cells (“GC-1/GC-2”) (**H**–**L**) were transfected with 500 nM of *KCNQ1OT1* siRNA (“seq1/seq2”, two rounds, total 48h) or the scramble control siRNA (“si-C”, two rounds, total 48h), expression of *KCNQ1OT1* (**A** and **H**), *miR-9* (**B** and **I**) and *LMX1A mRNA* (**C** and **J**) were tested by qPCR assays; LMX1A and LMX1B protein expression was tested by Western blotting assay (**C** and **K**, LMX1A protein results were quantified); Cells were further cultured for the indicated time periods, cell viability (**D**) and proliferation (**E** and **L**) were tested by the listed assays; Cell migration and invasion were tested by “Transwell” (**F**) and “Matrigel Transwell” (**G**) assays respectively. For each assay, n=5. *P <0.05 *vs.* “si-C” cells. Experiments in this figure were repeated three times, and similar results were obtained. Bar=100 μm (**E**–**G**).

Next, the siRNA strategy was employed to knockdown *KCNQ1OT1*. Two siRNAs, targeting non-overlapping sequence of *KCNQ1OT1* (namely “seq1/seq2” [24]), were transfected to AGS cells. Analyzing *KCNQ1OT1* expression by qPCR confirmed that the applied siRNAs resulted in over 60-70% reduction of *KCNQ1OT1* expression in AGS cells (Figure 4A). Significantly, *KCNQ1OT1* siRNA induced *miR-9* upregulation (Figure 4B) and *LMX1A mRNA*/protein downregulation (Figure 4C). Importantly, *KCNQ1OT1*-silenced cells presented with increased cell viability (Figure 4D) and EdU staining (Figure 4E), as compared to cells with scramble control siRNA (“si-C”) (Figure 4D, 4E). AGS cell migration and invasion, tested by “Transwell” assay (Figure 4F) and “Matrigel Transwell” assay (Figure 4G), were augmented by *KCNQ1OT1* siRNAs as well.

In primary human GC cells (“GC-1/GC-2”), transfection with the *KCNQ1OT1* siRNA (“seq2”) similarly resulted in *KCNQ1OT1* downregulation (Figure 4H), *miR-9* upregulation (Figure 4I) and *LMX1A* downregulation (Figure 4J, 4K). Cell proliferation, tested by an EdU staining assay, was enhanced as well (Figure 4L). These results further confirmed a tumor-suppressive function by *KCNQ1OT1*, and KCNQ1OT1 siRNA promoted GC cell progression *in vitro*.

### In LMX1A-knockout AGS cells altering *KCNQ1OT1* expression fails to change cell viability and proliferation

**Figure 5:**
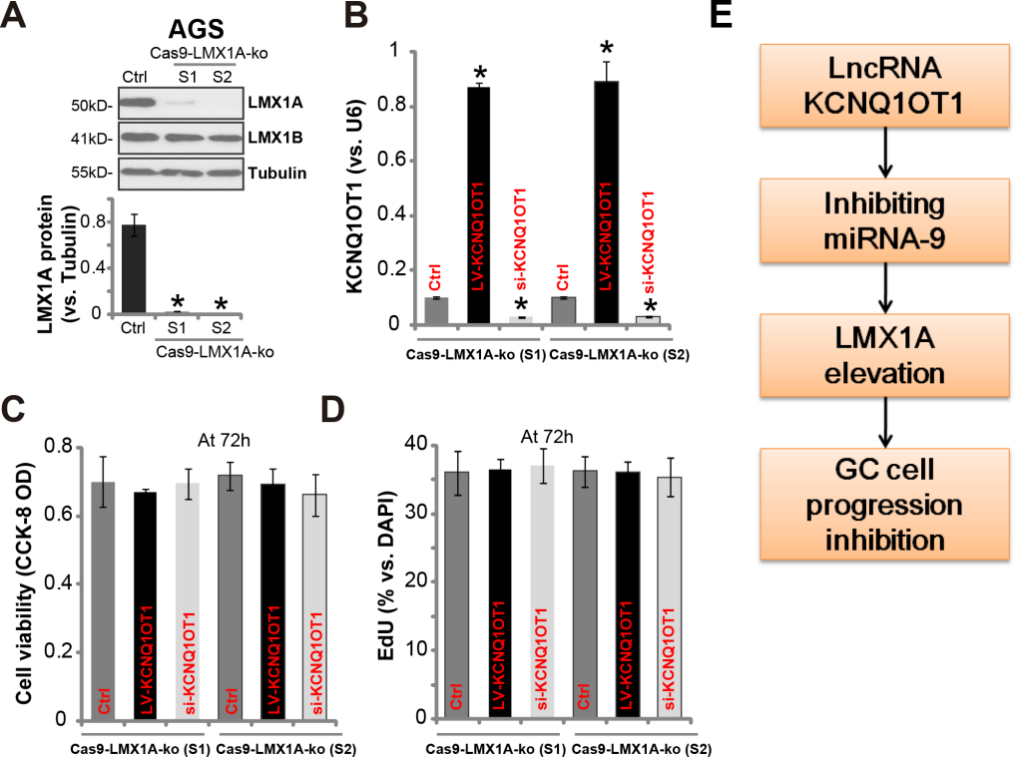
**In LMX1A-knockout AGS cells altering *KCNQ1OT1* expression fails to change cell viability and proliferation.** AGS cells were transfected with the lentiCRISPR/Cas9 LMX1A knockout constructs containing non-overlapping sgRNA sequences (“S1/S2”), following FACS sorting and puromycin selection two stable lines were obtained (“Cas9-LMX1A-ko”). LMX1A and LMX1B protein expression was tested (**A**, LMX1A protein results were quantified). LV-KCNQ1OT1 or *KCNQ1OT1* siRNA (500 nM) were transduced to the Cas9-LMX1A-ko AGS cells (“S1/S2”) for 72h, *KCNQ1OT1* expression (**B**), cell viability (**C**) and proliferation (**D**) were shown. (**E**) The proposed signaling cascade of this study. For each assay, n=5. *P <0.05 *vs.* “Ctrl” cells. Experiments in this figure were repeated three times, and similar results were obtained.

To verify that LMX1A is one target of *KCNQ1OT1*, we utilized the CRISPR/Cas9 gene-editing method to knockout LMX1A (see our previous study [7]). Two stable LMX1A knockout (“Cas9-LMX1A-ko”) AGS cell lines were established (namely “S1/S2”) [7]. Results in Figure 5A confirmed LMX1A protein depletion in the stable cells, with LMX1B unaffected (Figure 5A). LV-KCNQ1OT1 (see Figure 2) or *KCNQ1OT1* siRNA (“seq2”, see Figure 3) were tranduced to the Cas9-LMX1A-ko cells, both significantly altered *KCNQ1OT1* expression (Figure 5B). Significantly, neither LV-KCNQ1OT1 nor *KCNQ1OT1* siRNA altered viability (Figure 5C) and proliferation (EdU ratio, Figure 5D) in Cas9-LMX1A-ko AGS cells. Thus, in LMX1A-knockout AGS cells *KCNQ1OT1* did not change cell viability and proliferation, indicating that LMX1A is one target of *KCNQ1OT1*.

## DISCUSSION

Studies have proposed a possible role of *KCNQ1OT1* in carcinogenesis and progression of multiple cancers [21–23]. Recently, a high level of *KCNQ1OT1* was detected in lung cancer, promoting proliferation and invasion as well as chemoresistance of lung cancer cells [23, 25]. Similarly, *KCNQ1OT1* upregulation and mutation are associated with progression of hepatocellular carcinoma (HCC) and glioma [26, 27]. Feng et al*.,* reported that *KCNQ1OT1* upregulation in breast cancer is important for cancer cell growth *in vitro* and *in vivo* [21]. Li et al., however demonstrated that KCNQ1OT1 downregulation attenuated myocardial ischemia/reperfusion injury from acute myocardial infarction, possibly by regulating adiponectin receptor 1 (AdipoR1)-p38 /NF-κB signaling cascade [28]. Here, we show that *KCNQ1OT1* expression is downregulated in human GC tissues, which correlated with *miR-9* upregulation.

Very recent studies have proposed that *KCNQ1OT1* can function as a ceRNA. In HCC cells, *KCNQ1OT1* sponges *miR-504* to regulate the expression of *miR-504* target cyclin-dependent kinase 16 (CDK16) [29]. Furthermore, *KCNQ1OT1* directly sponges *miR-370* in glioma cells [26]. *KCNQ1OT1* induces IL-10 expression and macrophage polarization by sponging *miR-21a* [30]. It promoted caspase-1 activation via inhibiting *miR-214* [24]. We here propose that *KCNQ1OT1* could act as a ceRNA to sponge *miR-9* in GC cells, regulating LMX1A expression and GC cell functions.

In AGS cells and primary human GC cells, forced overexpression of *KCNQ1OT1* induced potent *miR-9* downregulation but LMX1A upregulation. Contrarily, *KCNQ1OT1* silencing, by two non-overlapping siRNAs, induced *miR-9* accumulation and LMX1A downregulation. Significantly, GC cell progression, including survival, proliferation, migration and invasion, were inhibited by *KCNQ1OT1* overexpression, but enhanced with *KCNQ1OT1* silencing. More importantly, in LMX1A knockout AGC cells, *KCNQ1OT1* overexpression or silencing was completely ineffective. Thus, we propose that *KCNQ1OT1* inhibits GC cell progression via regulating miR-9-LMX1A expression (Figure 5E). Taken together, we conclude that *LncRNA* *KCNQ1OT1* inhibits GC cell progression via regulating *microRNA-9*-LMX1A expression.

## MATERIALS AND METHODS

### Chemicals and reagents

Puromycin, neomycin and polybrene were obtained from Sigma-Aldrich (St. Louis, Mo). All antibodies were purchased from Abcam (Cambridge, MA). RNA reagents, Lipofectamine 2000 and other transcription reagents were obtained from Invitrogen (Shanghai, China). All sequences and plasmids were provided by Genechem (Shanghai, China).

### Cell culture

AGS cells were cultured as previously described [7]. The primary human GC cells, derived from two independent written informed-consent GC patients (“GC-1” and “GC-2”), were cultured in the described medium [31]. The protocols were approved by the Ethics Board of Minhang Hospital, Fudan University (Ethics number: 2015-052, Principle Investigator, Dr. Feng Li), in according to Declaration of Helsinki.

### Human tissues

As described previously [7], fresh GC cancer tissues along with matched surrounding normal gastric epithelial tissues from twelve (12) primary informed-consent GC patients (all male, 41–67 years old, with no prior chemotherapy and radiotherapy) were acquired. Tissues were washed, minced, and homogenized before further analysis.

### qPCR assay

For each treatment, aliquots of 100 ng total RNAs were reverse transcribed into cDNA with Reverse Transcriptase M-MLV (Promega, Madison, WI). The quantitative reverse transcriptase PCR (qPCR) assay was performed by the previously-described protocol [7], using ^ΔΔ^Ct method for target *mRNA* quantification and *GAPDH* as the internal control. *KCNQ1OT1* and *miR-9* expression was normalized to *U6 RNA*. Primers for *miR-9*, *LMX1A*, *GAPDH* and *U6* were reported previously [7]. The primers for *KCNQ1OT1*, forward, 5′-CTTTGCAGCAACCTCCTTGT; reverse, 5′-TGGGGTGAGGGATCTGAA, were also reported early [32].

### Forced *KCNQ1OT1* overexpression

The PCR primer sets, 5′-GGGGTACCCCAGGTGACAAGGTGCAGGCGC and reverse primer: 5′-ACAGAGTTCCTCGTTGGGAGCTTGAAGATCTTC [32], were applied to amplify the upstream KCNQ1OT1 promoter region from GC cells, inserted into the hU6-MCS-Ubiquitin-EGFP-IRES-puromycin vector (GV248, Genechem). The construct, along with the lentiviral packaging constructs [7], were transfected to HEK-293 cells to generate *KCNQ1OT1* expression lentivirus (“LV-KCNQ1OT1”). The lentivirus was filtered, enriched and added to GC cells. When necessary, stable cells were selected by puromycin (5.0 μg/mL) for a total of 4-5 passages. Control cells were infected with control lentivirus with empty vector.

### *KCNQ1OT1* siRNA

GC cells were seeded at 50% confluence. Two different siRNAs (designed and verified by RIBOBIO, Guangzhou, China [24]) against non-overlapping sequence of *KCNQ1OT1* were individually transfected (siRNA concentration at 500 nM) by Lipofectamine 2000 reagent for 24h, repeated for a second round for another 24h (total 48h). *KCNQ1OT1* knockdown was confirmed by qPCR assay. Control cells were transfected with the scramble non-sense control siRNA (“si-C”).

### Luciferase reporter assay

As reported [7], the human *LMX1A* 3’-UTR with the putative binding sites of *miR-9* (see the sites at [7]) was amplified by PCR and then inserted into the firefly luciferase reporter vector, pGL4.13 (luc2/SV40) (Promega), at the XbaI site and downstream from the stop codon of the luciferase gene. AGS cells were plated at 1 × 10^5^ cells per well into six-well plates (60% confluence), transfected with pGL4.13 construct along with the Renilla luciferase reporter vector and pRL-SV40 (Promega). Following the indicated genetic treatments, *LMX1A* 3’-UTR luciferase activity value was tested, and results were normalized to that in the control cells.

### Cell viability assay

Cells were seeded onto the 96-well plates (3 × 10^3^ cells/ well) [7]. Cell viability was determined via the Cell Counting kit-8 (CCK-8, Dojindo Laboratories, Kumamoto, Japan), with optical density (OD) values measured at 570 nm.

### *In vitro* cell migration and invasion assays

As described [33], GC cells were seeded on “Transwell” upper chamber (at 3 × 4000 cells per chamber, BD Biosciences). The complete medium (with 10% FBS) was added to the lower compartments. After 24h the migrated cells on the lower surface were stained. Matrigel (Sigma) was added in the chamber surface when analyzing cell invasion. Five repeated views in each condition were included to calculate the average number of migrated/invasive cells.

### EdU assay

EdU (5-ethynyl-20-deoxyuridine) Apollo-567 Kit (RIBOBIO, Shanghai, China) was employed to test cell proliferation. Briefly, cells were seeded onto the twelve-well plates. Following treatment, EdU and DAPI dyes were added, and cells were incubated for additional 8h. Cell nuclei were then visualized under a fluorescent microscope. For each condition at least 500 cells in five random views were included to calculate EdU ratio (EdU/DAPI×100%).

### Apoptosis assays

Testing cell apoptosis, by the Annexin V FACS and TUNEL staining assay, was described in detail in our previous study [7].

### LMX1A knockout

The two different lentiCRISPR-GFP-puro LMX1A knockout constructs (see our previous study [7]) were independently transfected to AGS cells by Lipofectamine 2000. GFP-positive cells were FACS-sorted, cultured for another three weeks. Stable cells were achieved by puromycin selection, named as “Cas9-LMX1A-ko” cells.

**Western blotting**

Total cellular lysates were resolved by SDS-PAGE (10%) gels, transferred to a PVDF membrane. The latter was incubated with blocking buffer and following primary and secondary antibodies. ECL method was applied to detect the immuno-reactive bands.

### Statistical analyses

The GraphPad Prism software was employed for statistical analyses. All values were expressed as the mean ± standard deviation (SD). Repeated-measures analysis of variance (RMANOVA) followed by Dunnett’s post hoc test for multiple comparisons were applied to evaluate statistical significance. All differences were considered significant at *P* < 0.05. To determine significance between two treatment groups, the two-tailed t tests were carried out.
